# Staged resection and anastomosis for multisegmental post-intubation tracheal stenosis

**DOI:** 10.1093/icvts/ivaf099

**Published:** 2025-05-20

**Authors:** Azizollah Abbasi Dezfuoli, Mahdi Zargarani, Hasan Alhalboni, Fariba Ghorbani

**Affiliations:** Lung Transplantation Research Center, National Research Institute of Tuberculosis and Lung Diseases, Shahid Beheshti University of Medical Sciences, Tehran, Iran; Tracheal Diseases Research Center, National Research Institute of Tuberculosis and Lung Diseases, Shahid Beheshti University of Medical Sciences, Tehran, Iran; Tracheal Diseases Research Center, National Research Institute of Tuberculosis and Lung Diseases, Shahid Beheshti University of Medical Sciences, Tehran, Iran; Tracheal Diseases Research Center, National Research Institute of Tuberculosis and Lung Diseases, Shahid Beheshti University of Medical Sciences, Tehran, Iran

**Keywords:** case report, postintubation tracheal stenosis, multisegmental tracheal stenosis, resection and anastomosis

## Abstract

Postintubation tracheal stenoses typically involve a single segment of the trachea and are treated by resection and anastomosis. This report presents a rare case of tracheal stenosis affecting 2 distinct segments of the trachea. A 16-year-old girl experienced severe postintubation tracheal stenosis following 2 prolonged intubations. The patient underwent staged resection and anastomosis. The first operation addressed the distal stenosis (intrathoracic). After the failure of non-surgical methods to relieve the proximal stenosis, the second resection was performed. The patient recovered with no signs of airway stenosis or voice problems during a 6-month follow-up period.

## INTRODUCTION

Postintubation tracheal stenoses (PITS) typically involve a single segment of the trachea [1]. As Grillo, and others have shown, most PITS can be treated with resection and anastomosis, provided inappropriate interventions do not render them unresectable [2, 3]. However, multisegmental tracheal stenosis (MSTS) is rare (∼3.83% of PITS cases) and challenging to treat [4, 5]. Due to the extended length of airway involvement, a single-session resection and anastomosis are often not feasible. This report discusses the management of a rare patient with such a complex stenosis.

## CASE REPORT

**Figure 1:**
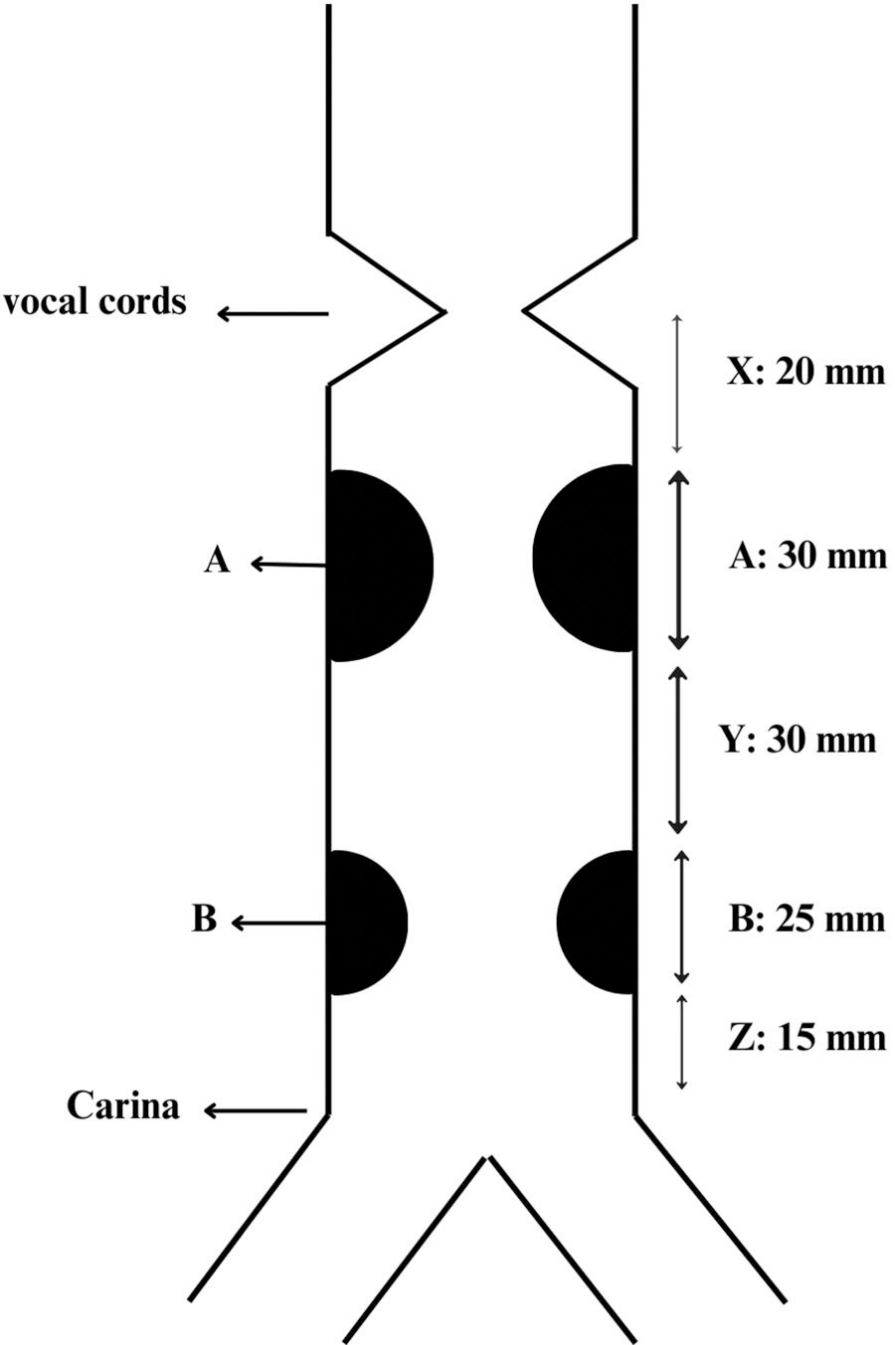
Schematic characteristics of stenosis. A: proximal stenosis; B: distal stenosis; X: distance of proximal stenosis to vocal cords; Y: normal trachea; Z: distance of distal stenosis to carina.

A 16-year-old girl attempted suicide by baclofen, requiring intubation for 20 days due to respiratory failure. After recovery, she developed progressive dyspnoea and signs of tracheal stenosis. Three weeks post-discharge, she presented with severe dyspnoea, pneumothorax and pneumomediastinum, necessitating a second 18-day intubation for antibiotic therapy and respiratory care. Following recovery, she was referred to Masih Daneshvari Hospital, where bronchoscopy revealed 2 separate stenotic segments (Fig. 1):

- Proximal: 20 mm below the vocal cords, measuring 30 mm in length, grade III (Cotton-Myer classification)
- Distal: 15 mm above the carina, measuring 25 mm in length, grade II

Five sessions of dilation by rigid bronchoscopy, size ranging from 5 to 9 (Brand Name: Storz), failed to provide long-term improvement. The time between dilatations varied from a few days to 2 months, with both areas of narrowing dilated during each bronchoscopic session.

**Figure 2:**
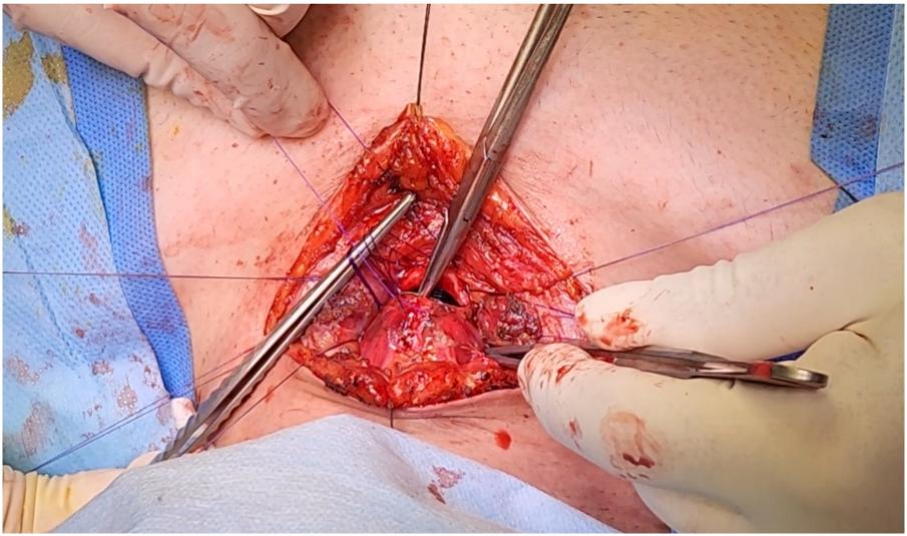
Continuous suturing of resected tracheal anastomosis with 3–0 polydioxanone sutures.

The distal stenosis was treated via a T-shaped incision and a partial sternotomy. Anterior tracheal release was performed from the first ring to the carina, and the membranous trachea was released only at the anastomosis site. After anterior tracheal release and resection of a 30-mm segment (Fig. 2), the tension on the anastomosis was moderate to high (subjectively).

The plan was to manage the proximal stenosis nonsurgically, but repeated rigid bronchoscopies and dilatations became less effective after 2 months. At this time, the distal anastomosis had healed without any stenosis, and all symptoms were due to the presence of the proximal stenosis. Finally, a second resection and anastomosis were performed for the proximal stenosis via a cervical incision. A 30-mm segment from the first ring downward was removed, and the trachea was anastomosed to the cricoid. In both operations, the releasing manoeuvre was limited to the anterior trachea, and the suturing technique was continuous polydioxanone suture 3–0. The larynx was also freed anteriorly from abnormal adhesions to facilitate the downward pull. Surprisingly, the tension in the second operation was felt to be less than that in the first.

The patient was extubated in the operating room, recovered well and exhibited normal breathing and voice (Video 1). No stent or bronchodilator was utilized in the postoperative management. The patient received antibiotics and prednisolone, initiated 6 days after the operation, at a dosage of 5 mg daily, which was continued for 1 month.

At 3 months post-surgery, bronchoscopy confirmed intact anastomoses with no residual stenosis. Normal breathing and voice quality were reported after 6 months.

## DISCUSSION

This case demonstrates the feasibility of staged resections for MSTS. Although Donahue and Grillo [3] reported success with repeated resections for recurrent stenosis, managing 2 distinct stenoses in separate operations remains under-reported.

In MSTS, we typically attempt to manage more severe stenosis through resection and anastomosis, while addressing the second stenosis using non-surgical methods. Stenosis of the intrathoracic airway poses greater challenges for resection and anastomosis compared to cervical stenosis; therefore, we prioritized the distal lesion for the initial operation. However, whenever the patient’s condition allows, we perform a resection for both stenoses [1, 5]. Successful resection and anastomosis of 2 separated segments within 2 months in this patient highlights the feasibility of such interventions. If inappropriate interventions like tracheostomy, laser or stents had been performed, the resection and anastomosis of 2 separate segments probably would not have been possible, reaffirming the resection anastomosis as the preferred treatment for PITS even when a large portion of the trachea is involved. Notably, despite significant tension in the first operation, the tension appeared to be less in the second, probably indicating that anastomotic tension decreases over time or due to the flexibility of the laryngotracheal skeleton. The intervals between 2 resection procedures and the decision-making process have been detailed in our published studies [1, 5]. The timing between the 2 resections depends on the patient’s condition and response to dilatation, so there is no set schedule. The schedule is tailored to each patient. Indeed, simultaneous resection of 2 stenoses is possible under certain conditions, primarily when the combined lengths of the stenotic and intervening normal trachea do not exceed half of the total tracheal length, allowing for a tension-free anastomosis. The distance between the stenoses should be short enough to facilitate a single resection without compromising vascular integrity. However, blood supply is a major concern, because the trachea relies on segmental arteries for perfusion. Excessive resection risks ischaemia at the anastomotic site, increasing the likelihood of complications like dehiscence or restenosis. Staged resections allow for healing and restoration of vascular integrity between operations, improving outcomes.

Most surgeons commonly use interrupted Vicryl sutures for tracheal anastomoses; however, we employ continuous suturing with polydioxanone sutures. We find this approach not only more technically efficient but also potentially superior in terms of clinical outcomes.

## Data Availability

The data underlying this article will be shared on reasonable request to the corresponding author.

## ABBREVIATIONS

MSTS: Multisegmental tracheal stenosis
PITS: Postintubation tracheal stenoses

## References

[1] Abbasidezfouli A, Shadmehr MB, Arab M et al Postintubation multisegmental tracheal stenosis: treatment and results. Ann Thorac Surg 2007;84:211–4. 17588414 10.1016/j.athoracsur.2007.03.050

[2] Grillo HC. Postintubation stenosis. In: Donahue DM (ed.) Surgery of the Trachea and Bronchi. Hamilton, London: BC Decker Inc., 2004, 301–40.

[3] Donahue DM, Grillo HC, Wain JC, Wright CD, Mathisen DJ. Reoperative tracheal resection and reconstruction for unsuccessful repair of postintubation stenosis. J Thorac Cardiovasc Surg 1997;114:934–9. 9434688 10.1016/S0022-5223(97)70007-2

[4] Zhu L, Gong X, Liu J et al Computational evaluation of surgical design for multisegmental complex congenital tracheal stenosis. Biomed Res Int 2020;2020:3509814. 32382545 10.1155/2020/3509814 PMC7191439

[5] Farzanegan R, Zangi M, Abbasidezfouli A et al Postintubation multisegmental tracheal stenosis: a 24-year experience. Ann Thorac Surg 2021;112:1101–8. 33232729 10.1016/j.athoracsur.2020.10.026

